# Oncological Outcomes, Long-Term Toxicities, Quality of Life and Sexual Health after Pencil-Beam Scanning Proton Therapy in Patients with Low-Grade Glioma

**DOI:** 10.3390/cancers15215287

**Published:** 2023-11-04

**Authors:** Jonas Willmann, Dominic Leiser, Damien Charles Weber

**Affiliations:** 1 Center for Proton Therapy, Paul Scherrer Institute, ETH Domain, 5232 Villigen, Switzerland; jonas.willmann@usz.ch (J.W.); dominic.leiser@psi.ch (D.L.); 2 Department of Radiation Oncology, University Hospital of Zurich, University of Zurich, 8006 Zurich, Switzerland; 3 Department of Radiation Oncology, Inselspital, Bern University Hospital, University of Bern, 3012 Bern, Switzerland

**Keywords:** low-grade glioma, proton therapy, pencil-beam scanning, oncological outcomes, patterns of failure, toxicity, quality of life, sexual health, patient-reported outcomes

## Abstract

**Simple Summary:**

Low-grade glioma are primary brain tumors mostly with IDH mutations, usually occurring in young patients, with a clinically good prognosis. Here, we analyzed patients with these tumors who were treated with proton therapy at our institution from 1999 to 2022. Among 89 patients included in our study, a favorable long term overall survival rate of 89% at four years was observed. Severe treatment-related side effects were rare and of low-grade. The global quality of life remained constant and comparable to the general population during proton therapy and during the first six years of follow-up thereafter. Sexual satisfaction as a domain of sexual health was comparable to a reference population. These results indicate that proton therapy for patients with low-grade glioma results in favorable oncological and patient-reported outcomes.

**Abstract:**

Purpose: To assess oncological outcomes, toxicities, quality of life (QoL) and sexual health (SH) of low-grade glioma (LGG) patients treated with pencil-beam scanning proton therapy (PBS-PT). Material and methods: We retrospectively analyzed 89 patients with LGG (Neurofibromatosis type 1; *n* = 4 (4.5%) patients) treated with PBS-PT (median dose 54 Gy (RBE)) from 1999 to 2022 at our institution. QoL was prospectively assessed during PBS-PT and yearly during follow-up from 2015 to 2023, while a cross-sectional exploration of SH was conducted in 2023. Results: Most LGGs (*n* = 58; 65.2%) were CNS WHO grade 2 and approximately half (*n* = 43; 48.3%) were located in the vicinity of the visual apparatus/thalamus. After a median follow-up of 50.2 months, 24 (27%) patients presented with treatment failures and most of these (*n* = 17/24; 70.8%) were salvaged. The 4-year overall survival was 89.1%. Only 2 (2.2%) and 1 (1.1%) patients presented with CTCAE grade 4 and 3 late radiation-induced toxicity, respectively. No grade 5 late adverse event was observed. The global health as a domain of QoL remained stable and comparable to the reference values during PBS-PT and for six years thereafter. Sexual satisfaction was comparable to the normative population. Conclusions: LGG patients treated with PBS-PT achieved excellent long-term survival and tumor control, with exceptionally low rates of high-grade late toxicity, and favorable QoL and SH.

## 1. Introduction

Gliomas are primary brain tumors that arise from glial cells. Since the late 1990s, the classification of gliomas was based on their microscopic appearance only, i.e., histology. Gliomas were and are still graded from WHO grade 1 to 4, with grade 1 and 2 representing low-grade glioma (LGG) [1]. LGGs tend to be slower growing and have a more favorable prognosis as compared to high-grade glioma. Since 2016, gliomas are further classified based on genetic alterations, in addition to histological analysis [2]. The most important diagnostic characteristics are the presence or absence of 1p/9q deletion and mutations of isocitrate dehydrogenase (IDH). The 2021 5th WHO Central Nervous System (CNS) classification introduced new tumor types and subtypes based on novel diagnostic technologies such as DNA methylome profiling [3].

LGGs most commonly occur in younger patients aged between 20 and 40 years [4]. Most of these patients will achieve substantial long survival, in many instances for well over a decade. Due to the indolent course of disease, treatment options need to be weighted carefully, considering not only tumor control but also potential late treatment-related side effects and the impact of these on the patients’ quality of life (QoL).

Surgery, radiotherapy, and chemotherapy may be administered to treat patients with LGG [5], and more recent targeted therapies have been introduced [6]. The role of each treatment modality, their sequence and timing remain controversial and is dependent on the type of LGG as well as patient characteristics and tumor-induced symptoms. Conventional radiotherapy with photons is among the treatment options for many patients with LGG and may improve survival and alleviate symptoms [7,8,9,10]. However, radiotherapy can adversely affect neurocognitive function, which is particularly concerning in younger patients with favorable outcomes, who may live with these side effects for years. Such side effects are often directly dependent on the dose delivered to healthy tissue adjacent to the target, i.e., the tumor and/or resection cavity after surgery.

Proton therapy (PT) is an alternative to conventional photon radiotherapy for the treatment of LGGs [11]. Compared with photon radiotherapy delivered for CNS tumors, PT reduces the brain integral dose and essentially eliminates the exit dose behind the target volume, without compromising tumor coverage, due to the rapid dose fall-off at the Bragg peak. Therefore, PT has the potential to reduce side effects and prevent neurocognitive decline [12]. Pencil-beam scanning proton therapy (PBS-PT) is a more conformal technique in comparison with the older passive-scattered proton therapy.

To date, there are only a limited number of studies on patients with LGG after treatment with PT with sufficiently long follow-up and rigorous reporting of long-term outcomes and side effects [13,14,15,16]. A prospective randomized phase 2 trial, comparing proton therapy to intensity-modulated radiotherapy (IMRT) with photons in patients with low- to intermediate-grade gliomas, is currently ongoing (NCT03180502), but study completion is expected only in 2030.

Besides side effects, which are physician-scored in most instances, patient-reported outcomes have gained clinical significance in recent years [17]. Frequently used instruments to measure the patient-reported outcomes of health-related quality of life (QoL) in brain tumor patients include the patient questionnaires European Organisation for Research and Treatment of Cancer (EORTC) Quality of Life Questionnaire (QLQ) Core 30 (C30) and Brain 20 (BN20) [18]. Sexual dysfunction as a domain of QoL has been recognized as a neglected concern in young brain tumor patients such as those with LGG [19]. The EORTC has recently validated the EORTC QLQ-SH22 questionnaire to assess the sexual health of patients with cancer at different treatment stages and during survivorship for clinical trials and for clinical practice [20].

In order to guide treatment decisions for patients with LGG, we analyzed oncological outcomes, long-term toxicities, QoL and SH in patients with LGG treated with PBS-PT at the Center for Proton Therapy (CPT)/Paul Scherrer Institute (PSI). The PSI has pioneered the clinical implementation of PBS-PT and thus has a large cohort of LGG patients with homogeneous treatment characteristics and an exceptionally long follow-up.

## 2. Materials and Methods

### 2.1. Patients

Eligible patients for this analysis were those with LGG, i.e., WHO CNS grade 1 or 2, pathologically confirmed or radiologically suspected, in the brain and treated with PBS-PT at PSI between January 1999 and July 2022. As during the study period of more than two decades, the definition of LGG according to the WHO has changed multiple times [1,2,3,21]—incorporating genetic alterations, in addition to histological features—we included patients if their tumor was classified as LGG at the time of treatment according to the most recent classification. No age restrictions applied. Patients with spinal LGG or treated with re-irradiation were excluded from this analysis.

### 2.2. Study Design

**Figure 1:**
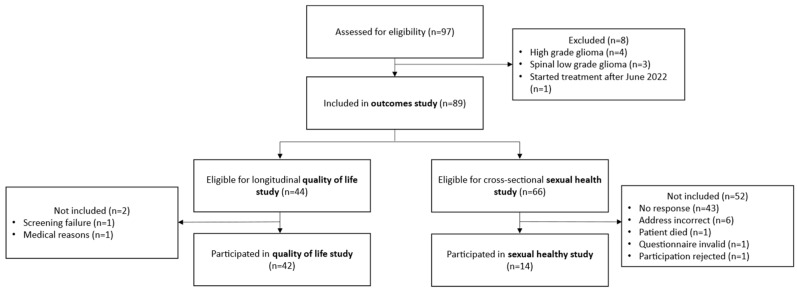
CONSORT diagram.

This study included a retrospective analysis of oncological outcomes and toxicities, as well as a prospective longitudinal analysis of QoL and a cross-sectional analysis of SH. The CONSORT diagram outlines the allocation of patients to the different analyses (Figure 1).

For the retrospective analysis of oncological outcomes, patients with LGG were included unless they rejected a general consent to use data for research purposes.

For the prospective longitudinal QoL study, patients at least 18 years of age at the start of PBS-PT were eligible, starting from June 2015 when PSI introduced the QoL questionnaire into routine clinical practice. The EORTC QLQ-C30 questionnaire, which includes 30 items to cover different aspects of QoL in cancer patients [22], and the EORTC QLQ-BN20 module, which includes 20 brain tumor-specific items [23], were employed to assess QoL before, during and at the end of PBS-PT, and yearly thereafter, as reported by the patient.

For the cross-sectional SH study, patients were eligible if they were alive at the time of the survey and at least 18 years of age at 1 January 2023. Eligible patients were contacted by letter and informed about the SH study from March to June 2023. For the assessment of SH, the EORTC QLQ-SH22 questionnaire was applied, using the patient’s self-assessment [20].

The study received approval from the cantonal ethics commission (EKNZ 2023-00184, 2023-00262).

### 2.3. Treatment and Follow-Up

Patient immobilization was achieved by employing a combination of a body cast and a vacuum-assisted bite-block system or thermoplastic mask. The prone position was utilized for immobilizing the patients, and their positioning was verified before each treatment fraction. All patients underwent 3D planning, which involved the co-registration of CT and MRI scans. Proton doses in our study were expressed in terms of Gray (Gy) relative biological effectiveness (RBE) using a factor of 1.1 compared to that of Co-60 [24]. Treatment was delivered in all patients using PBS-PT, utilizing energy-degraded beams from a 590 MeV cyclotron until 2005, and subsequently, a dedicated 250 MeV cyclotron was employed. This PBS-PT technique allows for the application of multiple, individually weighted proton pencil beams, which are typically evenly distributed on a three-dimensional grid. The optimization is performed using single-field uniform dose (SFUD) and intensity-modulated proton therapy (IMPT) modes.

A follow-up database was prospectively managed by the study and research office at CPT/PSI, with clinical evaluations including neuroendocrine, ophthalmologic, and auditory evaluations and radiographic imaging with MRI scans of the brain at regular intervals after treatment. Generally, during the initial two to three years, imaging follow-up examinations were conducted every three to six months, and annually thereafter.

### 2.4. Statistical Analysis

Descriptive statistics were utilized to describe continuous patient and treatment data variables, using median and interquartile range (IQR), and absolute counts and percentages for categorical data.

Overall survival (OS) and progression-free survival (PFS) were measured from the end of PBS-PT. Patterns of failure were assessed by comparing the location of the recurrent or progressive tumor on MRI in relation to the isodose lines from the initial PBS-PT treatment plan. Failures overlapping greater than the 80% isodose line were classified as in-field, overlapping the 80–20% isodose line as marginal, and others were classified as out-of-field failure.

Toxicities were graded according to the Common Terminology Criteria for Adverse Events (CTCAE) Version 5.0 and classified as acute (during and within 3 months after treatment) and late (more than 3 months after treatment) toxicity.

Univariate Cox regressions were employed to determine which clinical variables were associated with OS. The univariate analysis considered factors such as age at PBS-PT, CNS WHO grade (1; 2), location (frontal/temporal; visual apparatus/thalamic; other), indication (primary, definitive; primary, postoperative; recurrence/progression, definitive; recurrence/progression, postoperative), initial seizures (yes; no), and any initial symptoms (yes; no). Due to the small number of events, no multivariate analysis was performed.

The EORTC QLQ-C30 questionnaire and BN20 module QoL data, scored according to the EORTC manual, were assessed descriptively, showing the mean values of the scores over time at 9 time points (before, during and at the end of PBS-PT, and yearly thereafter until the sixth year). All scales and single-item measures range from scores 0 to 100. A high scale score represents a higher response level: for a functional scale a high score represents a high level of functioning, for the global QoL a high score represents a high QoL, but for a symptom scale a high score represents a high level of symptomatology. Results for selected items were compared with reference values of the general population in European countries [25].

The SH scores of the EORTC QLQ-SH22 questionnaire were calculated according to the manual by EORTC and reported descriptively with mean score values. The questionnaire includes 22 items, of which 2 are gender-specific; the remainder are gender-independent. All multi-item scales and single-item measures range from scores 0 to 100. A high score represents a high level of symptomatology or problems (i.e., suboptimal SH). As reference values from comparison with our results, we utilized mean values from cancer patients with no evidence of disease in the validation study, who we hypothesized to be comparable as all participants in our SH study were found to have controlled or stable disease as of their last available imaging [20]. Due to the limited number of participants in the SH study, the association of patient characteristics with SH scores was not evaluated.

A *p*-value of less than 0.05 was used as the threshold for statistical significance. All statistical analyses were performed in R (v4.1.2 x64, the R Foundation for Statistical Computing, Vienna, Austria, 2022) or SPSS (SPSS v28; IBM, Armonk, NY, USA).

## 3. Results

### 3.1. Patient and Treatment Characteristics

The study included a total of 89 LGG patients who were treated with PBS-PT at the CPT/PSI. Patient, tumor, and treatment characteristics are outlined in Table 1. The patients’ median age was 25.4 years (interquartile range [IQR] 12.3 to 38.1 years); 55 patients were above 18 years old at the time of proton therapy. Forty-nine (55.1%) patients were female. The most commonly treated LGG subtypes were astrocytoma (30.3%, *n* = 27), pilocytic astrocytoma (23.6%, *n* = 21) and oligodendroglioma (23.6%, *n* = 21). The majority of the tumors, 58 (65.2%), were classified as CNS WHO grade 2, while 17 (19.1%) were WHO CNS grade 1, and 14 (15.7%) were either unknown or diagnosed radiographically. Tumors were found to be distributed across different brain locations, with the largest group, 43 (48.3%) in the visual apparatus or thalamic area, followed by 31 (34.8%) in the frontotemporal region, and 15 (16.9%) in other parts of the brain. Tumors were evenly distributed in terms of laterality, with 37 (41.6%) centrally located, 27 (30.3%) on the right and 25 (28.1%) on the left hemisphere.

The isocitrate dehydrogenase 1 (IDH1) gene mutation status was available in 51 (57.3%) patients. Among those, 47 (92.2%, *n* = 47/51) patients had IDH mutations, while four (7.8%, *n* = 4/51) of the evaluable patients presented with the IDH wild type. The 1p19q deletion status was known in 35 (39.3%) patients, 17 of whom (48.6%, *n* = 17/35) had a co-deletion, while 18 (51.4%, *n* = 18/35) had a wild type variant. Only four patients (4.5%) had neurofibromatosis type 1.

For the confirmation of pathology, the majority, 61 (68.5%) patients were diagnosed through surgery, compared with 18 (20.2%) through biopsy—four of these were not assigned a WHO grade based on histopathological workup. A total of 10 patients (11.2%) were diagnosed radiographically. The study consisted of 46 (51.7%) primary treatments, and 43 (48.3%) treatments of recurrence or progression. The most common indication for PBS-PT was definitive recurrence/progression (37.1%, *n* = 33), followed by primary postoperative in (31.5%, *n* = 28), primary definitive in (20.2%, *n* = 18), and postoperative recurrence/progression in (11.2%, *n* = 10).

Twenty-four (27%) patients received chemotherapy before PBS-PT. Concomitant chemotherapy during PBS-PT was rarely administered (4.5%, *n* = 4); all of these cases received temozolomide. Following PBS-PT, 28 patients (31.5%) received adjuvant chemotherapy. The most common adjuvant chemotherapy regimen was procarbazine, CCNU (lomustine) und vincristine (PCV) (60.7%, *n* = 17/28), followed by temozolomide (35.7%, *n* = 10/28) and one other regimen (carboplatin and vincristine) received by one patient on a clinical trial (3.7%, *n* = 1/27).

### 3.2. Oncological Outcomes and Patterns of Failure

**Figure 2:**
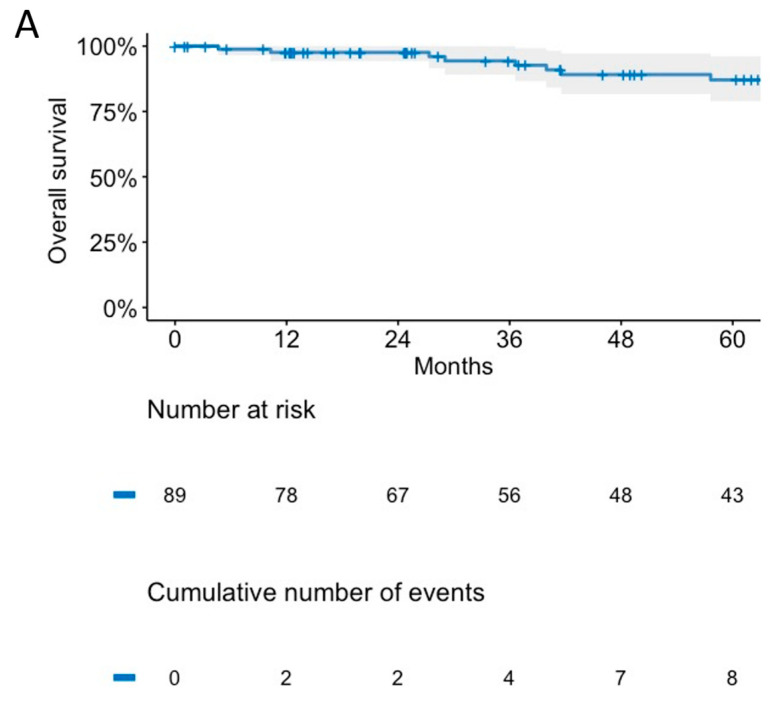

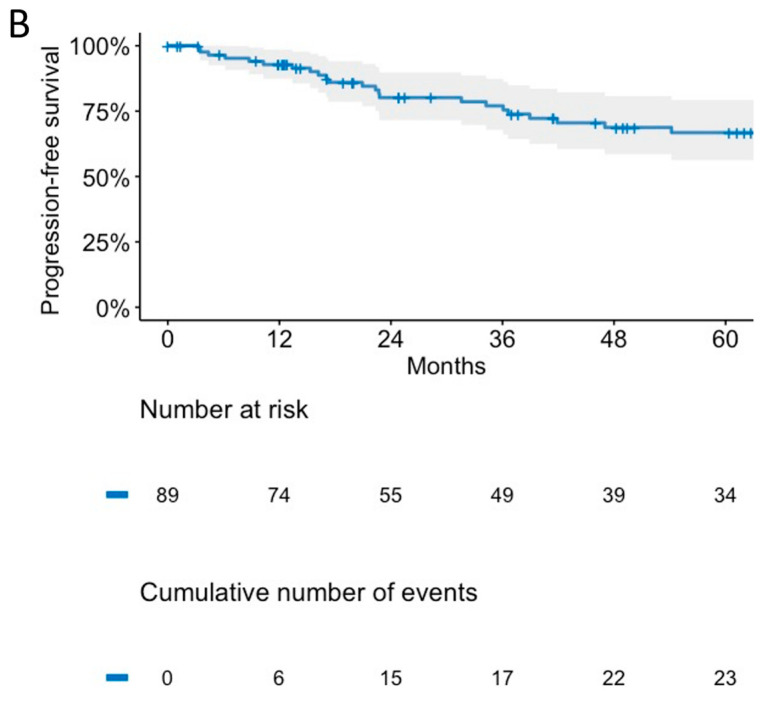
Kaplan–Meier curves for overall survival (**A**) and progression-free survival (**B**).

After a median follow-up of 50.2 months (interquartile range [IQR] 24.7–98.3), 15 (16.5%) deaths and 24 (27%) disease progression/recurrence events were observed. Among the 15 deaths, 8 (53.3%) were related to tumor progression and 3 (20%) to other causes, while for the 4 other patients the cause of death was unknown. In total, 26 patients experienced either death or disease progression. The OS rates at 3 and 4 years were 94.4% (95% confidence interval [CI] CI 89.1–100.0) and 89.1% (95% CI 81.7–97.2), respectively (Figure 2A). The PFS rate was 77.0% (95% CI 67.9–87.4) at 3 years, and 68.7% (95% CI 58.5–80.7) at 4 years (Figure 2B).

The local control rate at 4 years after PBS-PT was 71.1% (95% CI 61.0–82.9). The patterns of failure and post-progression treatments were analyzed. Twenty-four patients (27.0%) experienced treatment failure; sixteen (*n* = 16/24; 66.7%) of these documented failures were in-field, while for eight (*n* = 8/24; 33.3%), the type of failure could not be determined, due to missing follow-up imaging data. Among the patients who experienced treatment failure (*n* = 24), different salvage treatments (70.8%, *n* = 17/24) were delivered. The most common approach was surgery, utilized in eight (33.3%) of these cases. This was followed by systemic therapy, administered in six (25.0%) of the cases. Re-irradiation was the treatment of choice in only two (8.3%) of the cases, while combination therapy was used for one (4.2%) of the patients. Lastly, seven (29.2%) of the patients received the best supportive care following disease progression.

The exploratory univariate analysis failed to demonstrate statistically significant correlations between the covariates and OS, as detailed in Supplemental Table S1.

### 3.3. Toxicity

No patient experienced acute radiation-induced toxicity grade 3 or higher. However, 73 (82.0%) of the patients developed grade 1 or 2 acute toxicity, while 16 (18.0%) experienced no acute toxicity. In terms of late toxicity, 39 (43.8%) of the patients experienced any late toxicity, whereas in 50 (56.2%) patients no late toxicity was reported. Overall, only 3 (3.4%) patients experienced late toxicity of grade 3 or higher. Among these, 2 patients had grade 4 toxicities, and 1 had grade 3 toxicities. No grade 5 toxicities occurred. Both patients with grade 4 toxicities developed optic nerve disorders after PBS-PT, one of them in combination with grade 4 hypopituitarism.

One of the patients eventually developing grade 4 toxicity was initially treated with surgical debulking and PBS-PT to a total dose of 54 Gy (RBE) for a suprasellar pilomyxoid astrocytoma CNS WHO grade 2 and received adjuvant PCV chemotherapy. The patient’s vision deteriorated likely due to radiation necrosis and optic nerve atrophy, but no tumor progression was observed. MRI showed an inhomogeneously contrast-enhancing mass in the suprasellar region. Treatment with bevacizumab provided a mixed symptomatic response.

In the other case, the patient was treated with partial resection, followed by PBS-PT to a total dose of 54 Gy (RBE) and adjuvant PCV chemotherapy for a frontobasal oligodendroglioma WHO CNS grade 2. The patient developed both imaging changes consistent with radiation necrosis and tumor progression, leading to a deterioration in visual acuity and pituitary function. Treatment with bevacizumab, lomustine and corticosteroids did not result in significant visual improvements. The patient eventually presented with bilateral amaurosis due to radiation-induced optic nerve atrophy (grade 4) and panhypopituitarism (grade 4).

The third case with grade 3 toxicities was treated with definitive PBT-PT to a total dose of 60 Gy (RBE) to a right frontal oligodendroglioma in the late 1990s. One year after completion of treatment, the patient developed symptomatic grade 3 radiation necrosis with sensomotoric hemisyndrome and received corticosteroids for an extended period, combined with Pentoxifylline and vitamin E. Corticosteroids could be stopped after two years, when the patient’s symptoms improved. As of the time this manuscript was analyzed, the patient has remained free from disease progression and other high-grade toxicities for more than 25 years after treatment.

### 3.4. Quality of Life

The longitudinal QoL of patients with LGG before, during and after PBS-PT has been prospectively assessed at our center since 2015 using the EORTC QLQ C30 and BN20 questionnaires. Of 44 eligible patients, 42 (95.5%) participated in the QoL study (Figure 1).

Normative reference values for the general population were used to benchmark the QoL outcomes observed in our study [25]. Overall, the favorable QoL values were observed, which appeared numerically comparable to the reference values during PBS-PT and later on during follow-up.

**Figure 3:**
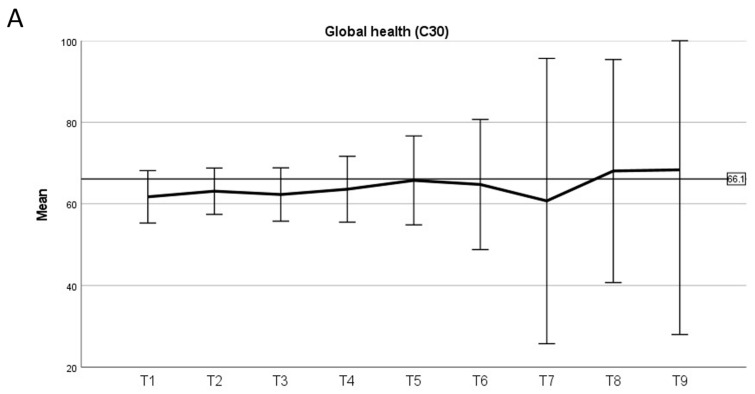

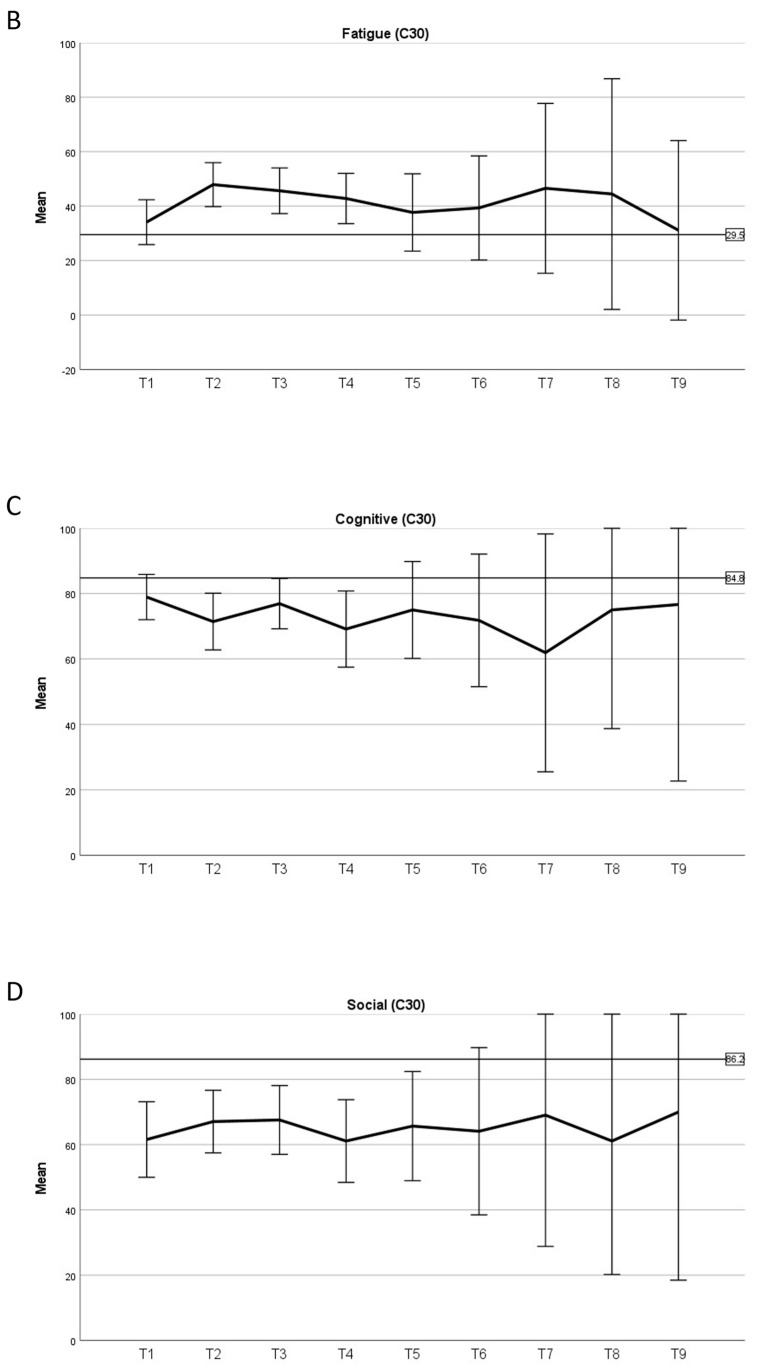
Quality of life: global health (**A**), fatigue (**B**), cognitive function (**C**) and social function (**D**) before (T1), during (T2) and the end (T3) of proton therapy and yearly thereafter (T4-9). Error bars denote 95% confidence interval. Black bold line indicates normative reference values for the general population as described by Nolte et al. [25].

The global health as a domain of QoL remained stable and comparable to the normative reference values during PT and during six years of follow-up thereafter (Figure 3A). Fatigue levels rose above the normative reference values during PBS-PT, and appeared comparable thereafter, while larger uncertainty due to limited sample size in later years of follow-up should be acknowledged (Figure 3B). Cognitive function was only slightly below the normative reference values, potentially decreasing slightly over the years of follow-up, while again firm conclusions are limited by small sample sizes (Figure 3C). The social functioning levels appeared lower than the reference values from the beginning of therapy, and remained at a consistently decreased level during follow-up (Figure 3D).

The results of other QoL scores from EORTC QLQ C30 and BN20 questionnaires are shown in the Supplementary Material (Figure S1).

### 3.5. Sexual Health

A cross-sectional SH study of LGG patients treated with PBS-PT was conducted from March to June 2023. Of 66 eligible patients, 14 (21.2%) participated (Figure 1). The median age of participants was 39 years (range, 22–59); 9 (64%) were females. A total of 5 (35.7%) and 3 (21.4%) participants had tumors in the frontal and temporal region, respectively; the rest were located in other brain areas. All participants had controlled or stable disease as of their last available imaging.

Our findings suggest comparable values for many SH scores between the current study and reference values from the validation study [20]. For sexual satisfaction, we observed a mean score of 44.86 (±22.77 standard deviation [SD]), compared to a mean score of 42.80 (±26.39 SD) in the reference population. A detailed overview of the results from our study and the reference population is detailed in Table 2.

## 4. Discussion

This study assessed the oncological outcomes, toxicity, QoL and SH in LGG patients treated with PBS-PT. Most patients had their tumors located in the visual/thalamic area, with astrocytoma being the most common subtype. We observed a 4-year overall survival rate of over 89% and a local control rate of approximately 70%. While most patients experienced mild acute toxicities, severe cases of late toxicities, mainly visual due to the main tumor localization, were exceedingly rare. The longitudinal QoL analysis indicated stable global health scores, increased fatigue during treatment, and a slight decline in cognitive function over time. Additionally, a separate assessment of SH suggested that the patients’ sexual satisfaction was on par with the general population’s reference values. These encouraging results highlight the potential of PBS-PT as a promising and well-tolerated treatment modality for patients with LGG who will live substantially longer than other brain tumor patients, reaffirming its position in the therapeutic landscape and offering a valuable option for preserving the QoL and SH.

With radiotherapy playing a crucial role in the treatment of LGG [10,26,27,28]—alone or in combination with surgery and/or chemotherapy—PT represents an interesting alternative treatment option aimed at decreasing long-term side effects while preserving optimal tumor control. Several studies have explored the outcomes of patients with LGG after treatment with PT (Table 3). In a prospective single-arm trial involving 20 patients with CNS WHO grade 2 glioma treated with passive-scattered PT (median follow-up of 5.1 years), treatment was well-tolerated, with no evidence of an overall decline in cognitive function or quality of life [16]. The 3-year progression-free survival rate was 85% and thus only slightly less than in our study. These findings confirm the minimal occurrence of higher-grade toxicity observed in our study. Another prospective study of 174 pediatric patients (≤21 years old) with LGG (70.1% WHO grade 1) treated with PT from 2007 to 2017 also revealed favorable clinical outcomes [29]. The 5-year actuarial rates of local control, progression-free survival, and overall survival were 85%, 84%, and 92%, respectively. Acute side effects were mainly nausea or vomiting (12.6%), with only 4% experiencing serious toxicities such as brainstem necrosis or radiation retinopathy. These observations confirm that PT may curtail acute toxicities, while effectively maintaining disease control, and align with our study’s findings, underscoring the efficacy and safety of PT in both pediatric and adult LGG cases. In a retrospective series involving 32 pediatric patients with LGG (59.4% CNS WHO grade 1) treated with PT between 1995 and 2007, encouraging results were observed, with 8-year overall survival rates reaching 100% and progression-free survival rates of 82.8% at the same time point [13]. PT demonstrated good clinical outcomes, with particular benefit in patients where the tumor location allowed increased sparing of critical brain regions. Although neurocognitive outcomes remained stable for most, younger children and those with significant irradiation to the left temporal lobe/hippocampus exhibited some declines.

The patterns of failure were analyzed in a retrospective analysis of 141 patients with CNS WHO grade I to II or IDH1-positive mutation grade III LGG treated with PT between 2005 and 2015 [30]. The majority of tumor recurrences occurred within the radiation field. The 5-year freedom from progression post-PT stood at 60.1%; 74% of the failures were observed in-field, only 12% were out-of-field, and another 12% had marginal failures. Strikingly, patients who experienced tumor recurrence after PT had a significantly reduced 5-year cumulative incidence of overall survival at 33%, in contrast to a 96% survival rate for those without recurrence. These patterns of failure are in line with observations from our study, and highlight the role of local tumor control to ensure optimal survival.

The main advantage of PT is the potential reduction in late radiation-induced side effects that detrimentally affect the QoL in LGG patients, many of whom are young and survive with their condition for many years. A systematic review on long-term effects of radiotherapy for patients with LGG, evaluating nine studies involving 2406 participants, suggested that radiotherapy might heighten the risk of long-term cognitive impairment, though the exact risk magnitude remains ambiguous [31]. Notably, younger patients undergoing conventional radiotherapy seemed more prone to neurocognitive issues. When comparing the combination of chemoradiotherapy versus radiotherapy alone, no difference in cognitive effects or quality of life were, however, discernible. By minimizing the dose to critical neurological structures, PT holds the potential to reduce long-term neurocognitive and quality-of-life decline. In a retrospective planning study, comparing the original PT plans of 74 pediatric patients with LGG to conventional photon radiotherapy plans, PT allowed for a marked reduction in doses to crucial brain structures and areas of neurogenesis [32]. It was noteworthy that contralaterally located structures benefited from the dose-sparing properties of PT.

Studies on long-term toxicities and QoL, with or without neurocognition, in patients with LGG after PT are scarce. In a prospective cohort study involving 20 patients with LGG treated with PT, the majority maintained stable cognitive and neuroendocrine function over a median follow-up of 6.8 years [33]. The study highlights that most long-term toxicities manifested within two years post-treatment. Six patients developed neuroendocrine deficiencies, especially those receiving higher doses to the hypothalamus–pituitary axis. In a longitudinal study examining the neurocognitive outcomes of PT in 20 patients with LGG over five years, results showed general stability in cognitive function [34]. Interestingly, patients with tumors in the left hemisphere initially displayed more impairment in verbal measures compared to those with tumors in the right hemisphere, yet over time, they exhibited notable improvements in verbal memory. These findings emphasize the favorable profile of PT in preserving cognitive function and its low rate of higher-grade late toxicities, while also underscoring the importance of comprehensive neuropsychological assessments.

SH is a neglected concern in young patients with brain tumors in general, and with LGG in particular. According to a recent systematic review of the limited literature available on SH in neurooncological patients—three studies were found that covered 124 brain tumor patients, of which 62 (50%) had LGG—the incidence of sexual dysfunction ranged from 44 to 63% [19]. The potentially high prevalence of sexual dysfunction among LGG patients, coupled with their young age and extended life expectancy, emphasizes the need for more robust investigations of SH and tailored therapeutic strategies to improve long-term QoL. To the best of our knowledge, no published study has systematically investigated SH in LGG patients after PT.

In a cross-sectional study assessing the potential impact of surgery on the SH of LGG patients, 32 individuals who underwent surgery without radiotherapy and resumed normal social and professional lives were evaluated using the Arizona Sexual Experiences Scale (ASEX) and additional subjective assessments [35]. The majority, or 53% of the patients, reported changes in their postoperative sexual experiences, with a predominant 88% noting deterioration. More women (60%) than men (29%) reported sexual dysfunction. The study found that right-sided surgical resections were linked to greater difficulties in achieving orgasm than left-sided operations. Additionally, male patients who had temporal lobe resections encountered more significant declines in sexual drive and arousal, with these reductions being more pronounced than in female counterparts. Notably, men continuing with antiepileptic drugs post right-sided resection had higher overall ASEX scores compared to women. The findings highlight the prevalence of postoperative sexual dysfunction in LGG patients, emphasizing the importance of routinely addressing SH before and after surgery.

In another cross-sectional study exploring the impact of brain tumors on sexual function, 46 patients with brain tumors were assessed for their perceptions of their sexual well-being using a customized questionnaire, and psychological assessments using various tools, including the Hospital Anxiety and Depression Scale and the EORTC QLQ-C30 questionnaire [36]. About half of the patients had LGG, but the administered treatment, including radiotherapy, was not reported. The results revealed that 58% of the patients experienced sexual disturbances, with a notable 56% indicating a decline or absence of sexual desire as their primary concern. Only 15.2% of the participants were informed by their doctors about potential changes in their sexual sphere, with only 10.8% of this subset having specifically sought out such information. The study underscores the correlation between QoL and SH in brain tumor patients and underscores the need for clinicians to actively address patients’ sexual concerns, facilitating their disclosure of problems and ensuring they receive the necessary support. The third cross-sectional study assessed QoL and sexual dysfunction in 50 brain tumor patients—only nine (18%) of whom were diagnosed with LGG [37]. About half of the patients received radiotherapy in combination with surgery and chemotherapy. Sexual dysfunction rates were higher than previously noted, with 66% of sexually active women and 60% of men showing dysfunction.

Several limitations to our study should be acknowledged, primarily due to the retrospective assessment of oncological outcomes and toxicities, which can introduce potential biases and uncertainties, and certain events such as lower grade toxicities or tumor progressions may be underreported. Moreover, different WHO classifications were applied over the years and combined for our analysis, without an updated, central pathology review, which was not deemed feasible. Finally, small patient figures obviate correlative analysis of no patient’s characteristics and outcomes.

The strengths of our study lie in its prospective assessment of QoL and analysis of SH. Moreover, the study benefits from a long-term follow-up, which offers a deeper understanding of survival, tumor control and the late-onset effects of PBS-PT in LGG patients. This extended observation period enhances the validity of the outcomes observed. Few prior studies have shed light on SH in LGG patients, but their interpretability is hampered by the heterogeneity of participants and treatment, and cross-study comparison was rendered near-impossible as different tools were used to measure SH. Our study stands out given its homogeneity regarding patient and treatment characteristics, and as it uses the recently validated EORTC SH22 questionnaire. It may, thus, serve as a benchmark for future studies assessing SH in LGG patients and to compare the impact of different treatment modalities. Future studies should include larger patient groups and perform longitudinal assessment to determine changes over time, and correlate SH with patient and treatment characteristics. The longitudinal assessment of different QoL domains after PBS-PT in our study gives a unique insight into the long-term trajectory of these patient-reported outcomes and may, thus, inform shared decision making with patients.

## 5. Conclusions

Patients with LGG treated with PBS-PT achieved long-term tumor control and survival. Most patients who developed local tumor progression could be salvaged. The rates of high-grade late toxicities were exceptionally low in our cohort. QoL and SH as reported by patients in our study were favorable. PBS-PT may, thus, be a promising treatment option of LGG patients for whom radiotherapy is indicated.

## Figures and Tables

**Table 1:** Patient, tumor and treatment characteristics.

Variable	Overall (N = 89)
**Age**	
Median (IQR) in years	25.4 (12.3–38.1)
**Sex**	
female	49 (55.1%)
male	40 (44.9%)
**Subtype**	
Oligodendroglioma	21 (23.6%)
Astrocytoma	27 (30.3%)
Oligoastrocytoma	2 (2.2%)
Pilocytic	21 (23.6%)
Pilomyxoid	6 (6.7%)
Other/unknown	12 (13.5%)
**WHO grade**	
2	58 (65.2%)
1	17 (19.1%)
Unknown or radiographically diagnosed	14 (15.7%)
**Location**	
Frontotemporal	31 (34.8%)
Visual/thalamic	43 (48.3%)
Other	15 (16.9%)
**Laterality**	
Right	27 (30.3%)
Left	25 (28.1%)
Central	37 (41.6%)
**IDH status**	
Wild type	4 (4.5%)
Mutated	47 (52.8%)
Unknown	38 (42.7%)
**1p19q status**	
Wild type	18 (20.2%)
Co-deleted	17 (19.1%)
Unknown	54 (60.7%)
**Neurofibromatosis type 1**	
No	85 (95.5%)
Yes	4 (4.5%)
**Pathology confirmation**	
Radiological	10 (11.2%)
Biopsy	18 (20.2%)
Surgery	61 (68.5%)
**Tumor type**	
Primary	46 (51.7%)
Recurrence/progression	43 (48.3%)
**Treatment indication**	
Primary, definitive	18 (20.2%)
Primary, postoperative	28 (31.5%)
Recurrence/progression, definitive	33 (37.1%)
Recurrence/progression, postoperative	10 (11.2%)
**Radiotherapy dose**	
Median (interquartile range; range) in Gy (RBE)	54 (52.2–54; 46–64)
**Previous chemotherapy**	
No	65 (73.0%)
Yes	24 (27.0%)
**Concomitant chemotherapy**	
No	85 (95.5%)
Yes	4 (4.5%)
**Adjuvant chemotherapy**	
No	61 (68.5%)
Yes	28 (31.5%)

**Table 2:** Sexual health according to the EORTC QLQ-SH22 questionnaire in our study, compared with reference values of patients with no evidence of disease in the validation study by Greimel et al. [20].

	Current Study			Reference		
	Total	Mean	Standard Deviation	Total	Mean	Standard Deviation
Sexual satisfaction	14	44.86	22.77	152	42.80	26.39
Sexual pain	12	6.57	13.79	151	19.98	27.50
Importance of sexual activity	14	36.11	33.21	153	45.53	32.84
Decreased libido	14	41.67	35.18	153	46.84	36.56
Worry incontinence	14	13.89	26.43	154	17.97	29.80
Fatigue	14	36.11	33.21	151	37.97	35.70
Treatment effect on sexuality	13	30.56	33.21	152	51.32	39.47
Communication with professionals	14	91.67	20.72	151	17.44	29.02
Insecurity with partner	13	25.00	28.87	144	29.63	35.06
Confidence erection	5	25.00	31.92	55	39.39	37.46
Body image (male)	5	16.67	19.24	56	39.88	35.63
Vaginal dryness	9	37.50	37.53	88	46.21	39.93
Body image female	9	25.00	29.55	96	34.03	37.14

**Table 3:** Summary of selected prospective or large retrospective studies on PT for LGG patients.

First Author, Year	Design	Patients	Main Inclusion Criteria	Median Follow-Up (Years)	Outcomes	Toxicity
Shih, 2015 [16]	Prospective single-arm	20	LGG WHO grade 2 ≥19 years of age No baseline cognitive deficits to compromise neurocognitive assessment No prior cranial irradiation	5.1	PFS at 3 years 85%, at 5 years 40%	No changes in QoL, cognitive function Baseline neurocognitive impairment in 8 patients New endocrine dysfunction in 6 patients
Greenberger, 2014 [13]	Retrospective database	32	LGG WHO 1 and 2 Location in brain or spinal cord ≤21 years of age	7.6	PFS at 6 years 89.7%, at 8-year 82.8% OS at 8 years 100%	No significant declines in neurocognition Subgroup analysis indicated significant neurocognitive decline for young children (<7 years) and those with higher dose to the left temporal lobe/hippocampus
Indelicato, 2019 [29]	Prospective single-arm	174	LGG WHO 1 and 2 Location in brain or spinal cord ≤21 years of age	4.4	5-year actuarial local control 85% 5-year actuarial PFS 84% 5-year actuarial OS 92%	4% of patients with serious toxicities, including brainstem necrosis requiring corticosteroids, symptomatic vasculopathy, radiation retinopathy, epilepsy, and death from radiation-induced high-grade glioma Central hormone deficiency in 39 patients

## Data Availability

The data presented in this study are available on request from the corresponding author.

## Supplementary Materials

The following supporting information can be downloaded at: https://www.mdpi.com/article/10.3390/cancers15215287/s1, Figure S1: Quality of life: additional scores; Table S1: Univariate analysis of factors associated with overall survival.
