# Supplementary material for: Oncological Outcomes, Long-Term Toxicities, Quality of Life and Sexual Health after Pencil-Beam Scanning Proton Therapy in Patients with Low-Grade Glioma
Source: Cancers (Basel). 2023 Nov 4;15(21):5287. doi: 10.3390/cancers15215287 (PMC10649084; doi:10.3390/cancers15215287)
Supplement: Supplementary file 1 [file cancers-15-05287-s001.zip › cancers-2679318-supplementary.pdf]

cancers

MDPI

**Table S1**

Univariable analysis of factors associated with overall survival.

| | | | Hazard ratio (95% CI, p-value) |
|---|---|---|---|
| Age (years) | *Mean (SD)* | 26.7 (16.3) | 1.03 (1.00-1.06, p=0.050) |
| WHO CNS grade | *2* | 58 (65.2) | - |
| | *1* | 17 (19.1) | 0.38 (0.11-1.37, p=0.139) |
| Location | *Frontotemporal* | 31 (34.8) | - |
| | *Visual/thalamic* | 43 (48.3) | 0.28 (0.08-1.02, p=0.054) |
| | *Other* | 15 (16.9) | 1.23 (0.34-4.48, p=0.758) |
| Indication | *Primary, defitive* | 18 (20.2) | - |
| | *Primary, postoperative* | 28 (31.5) | 1.91 (0.26-14.21, p=0.528) |
| | *Recurrence/progression, definitive* | 33 (37.1) | 2.84 (0.60-13.45, p=0.189) |
| | *Recurrence/progression, postoperative* | 10 (11.2) | 2.91 (0.41-20.88, p=0.289) |
| Initial seizures | *No* | 55 (61.8) | - |
| | *Yes* | 34 (38.2) | 1.55 (0.54-4.40, p=0.415) |

**Figure S1**

Quality of life: additional scores.

*EORTC QLQ-C30*

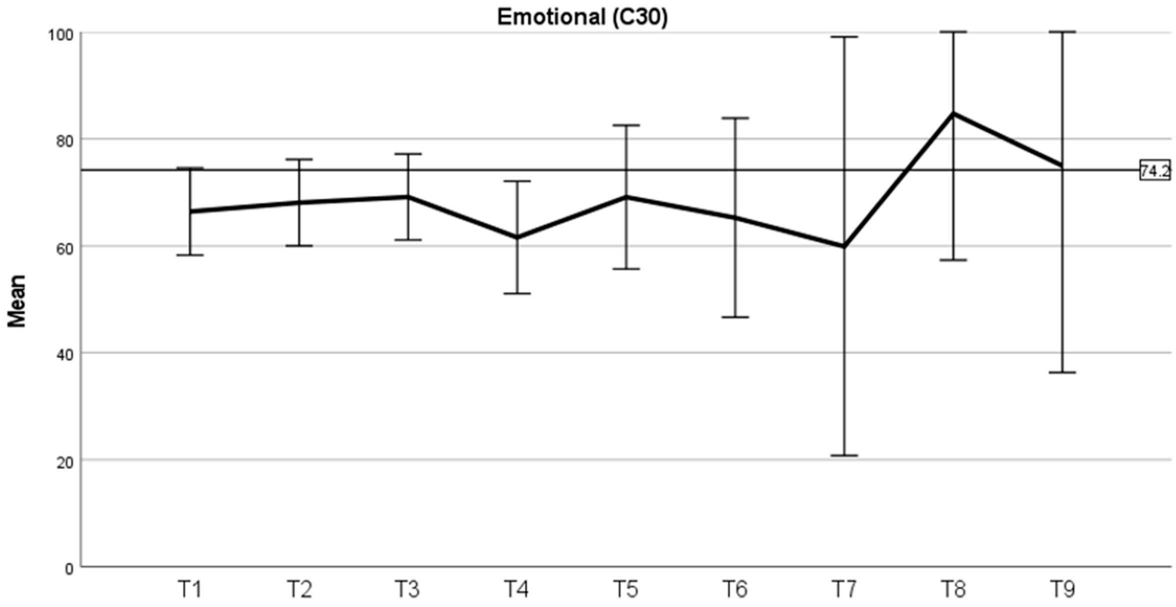
Emotional (C30)
Mean
0
20
40
60
80
100
74.2
T1
T2
T3
T4
T5
T6
T7
T8
T9

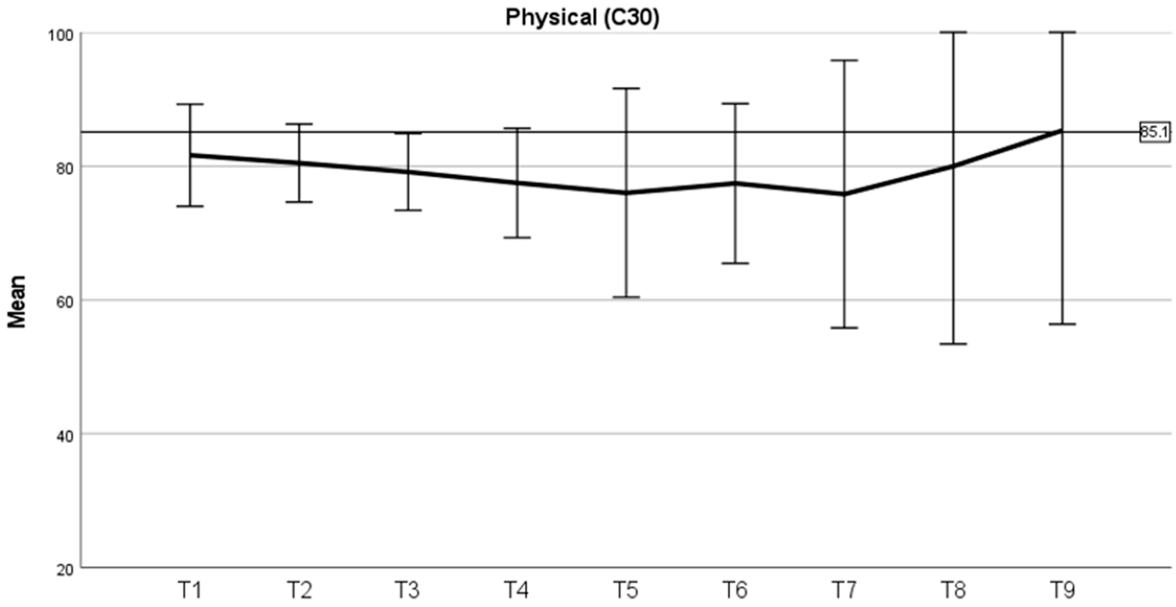
Physical (C30)
Mean
20
40
60
80
100
85.1
T1
T2
T3
T4
T5
T6
T7
T8
T9

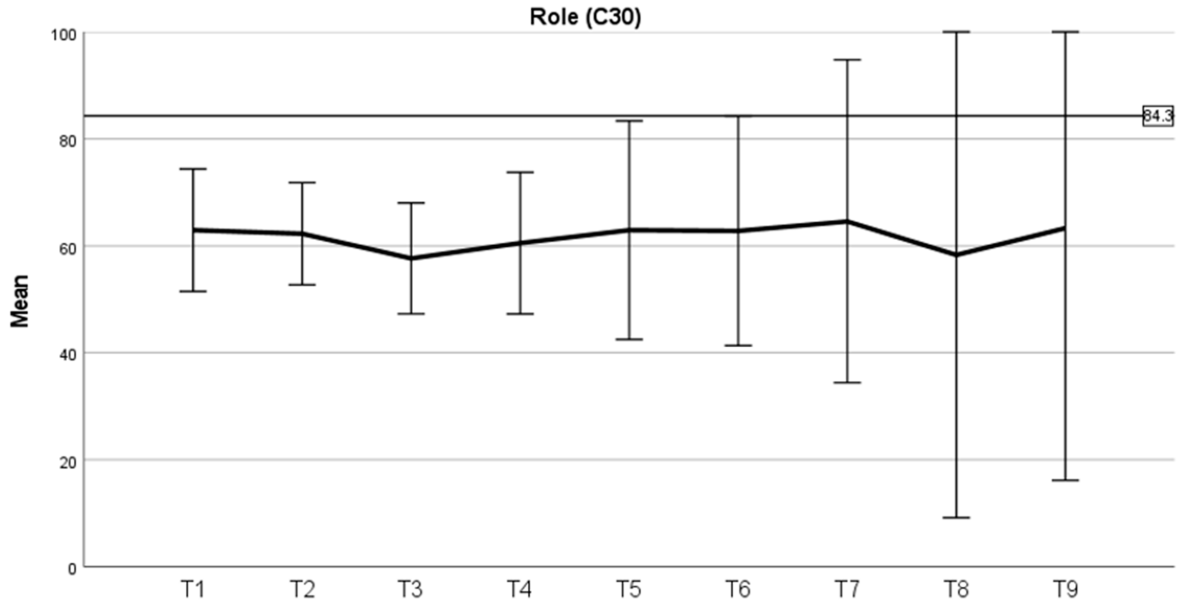
Role (C30)
100
80
60
40
20
0
Mean
84.3
T1
T2
T3
T4
T5
T6
T7
T8
T9

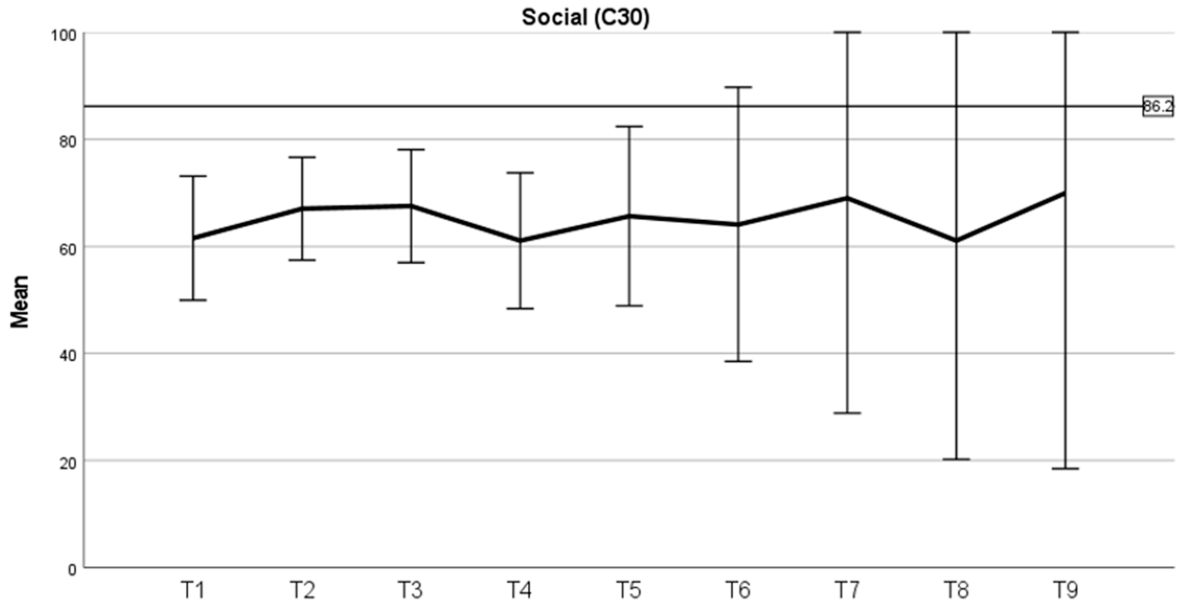
Social (C30)
100
80
60
40
20
0
Mean
86.2
T1
T2
T3
T4
T5
T6
T7
T8
T9

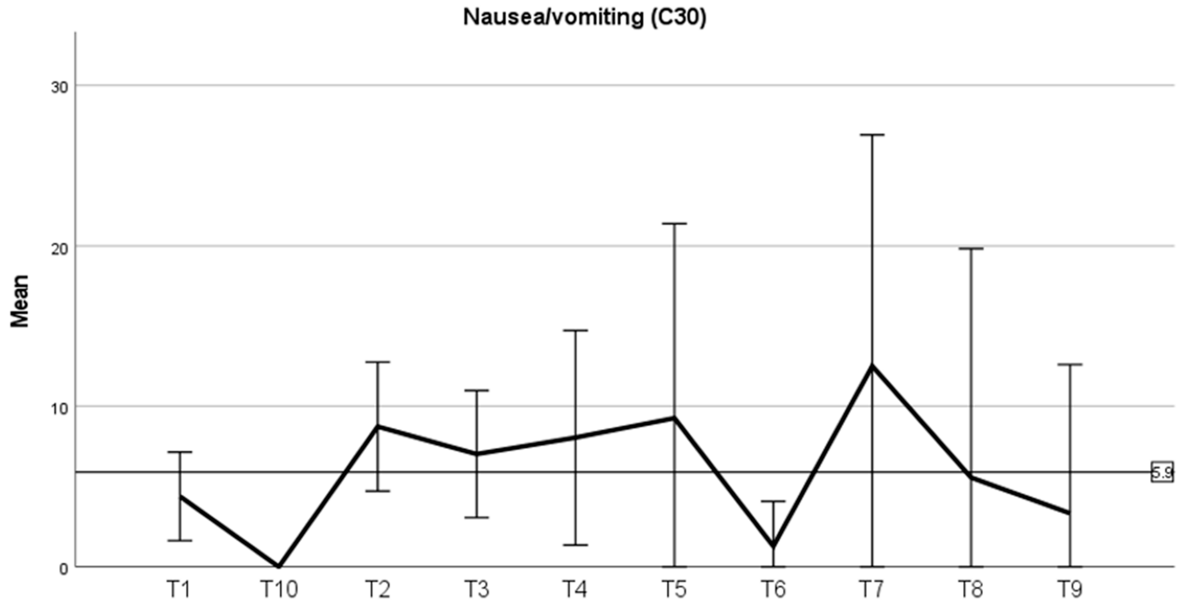
Nausea/vomiting (C30)
Mean
0
10
20
30
5.9
T1
T10
T2
T3
T4
T5
T6
T7
T8
T9

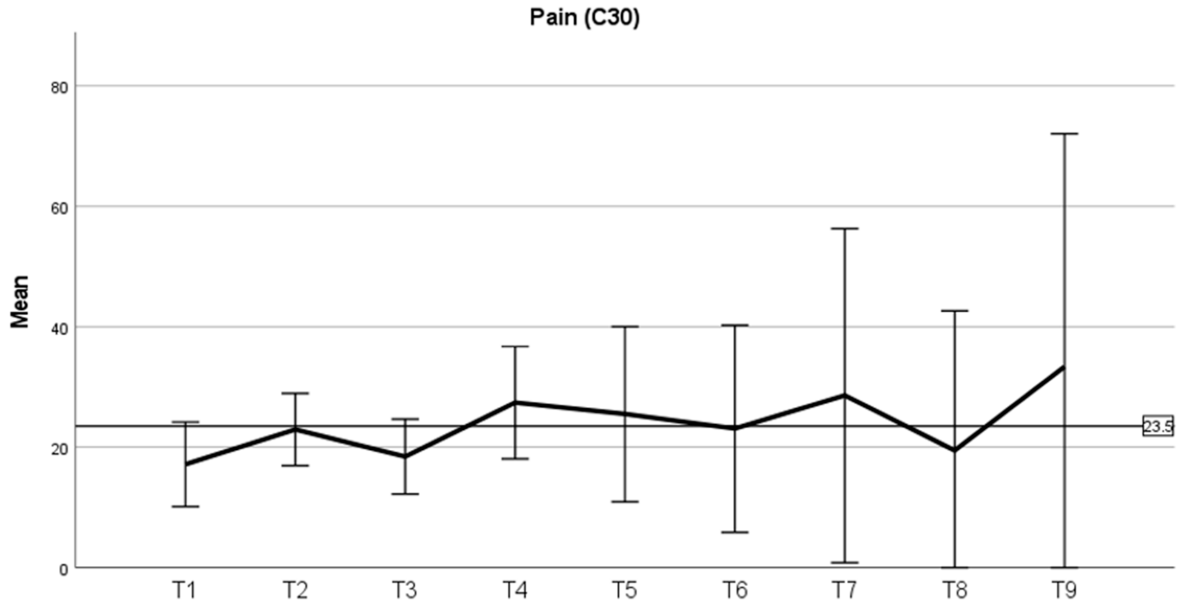
Pain (C30)
Mean
0
20
40
60
80
23.5
T1
T2
T3
T4
T5
T6
T7
T8
T9

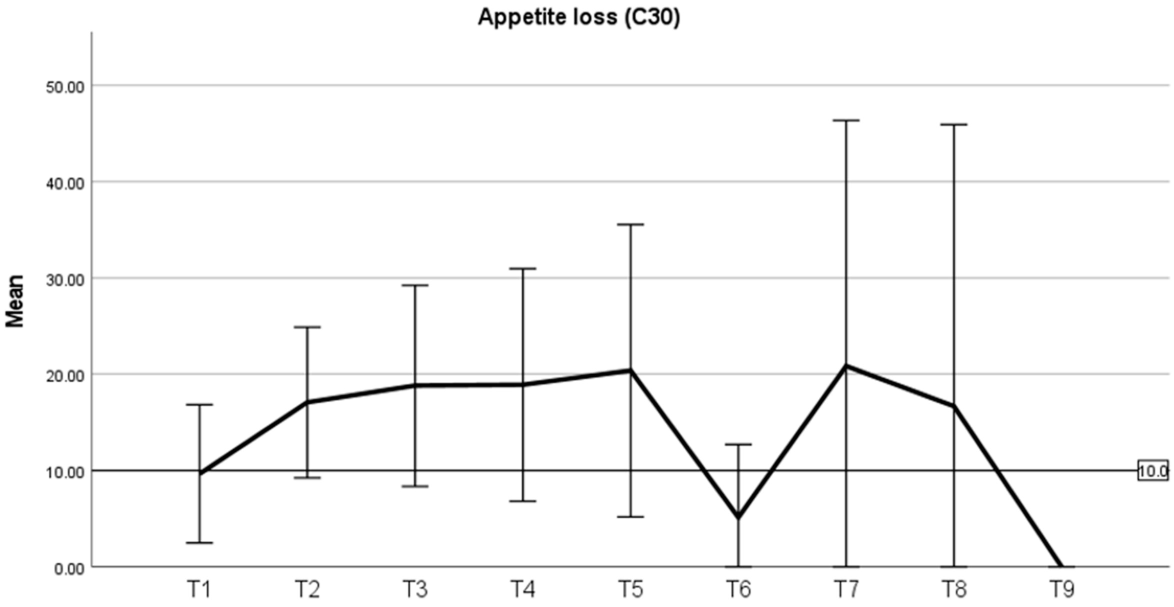
Appetite loss (C30)
Mean
50.00
40.00
30.00
20.00
10.00
0.00
10.0
T1
T2
T3
T4
T5
T6
T7
T8
T9

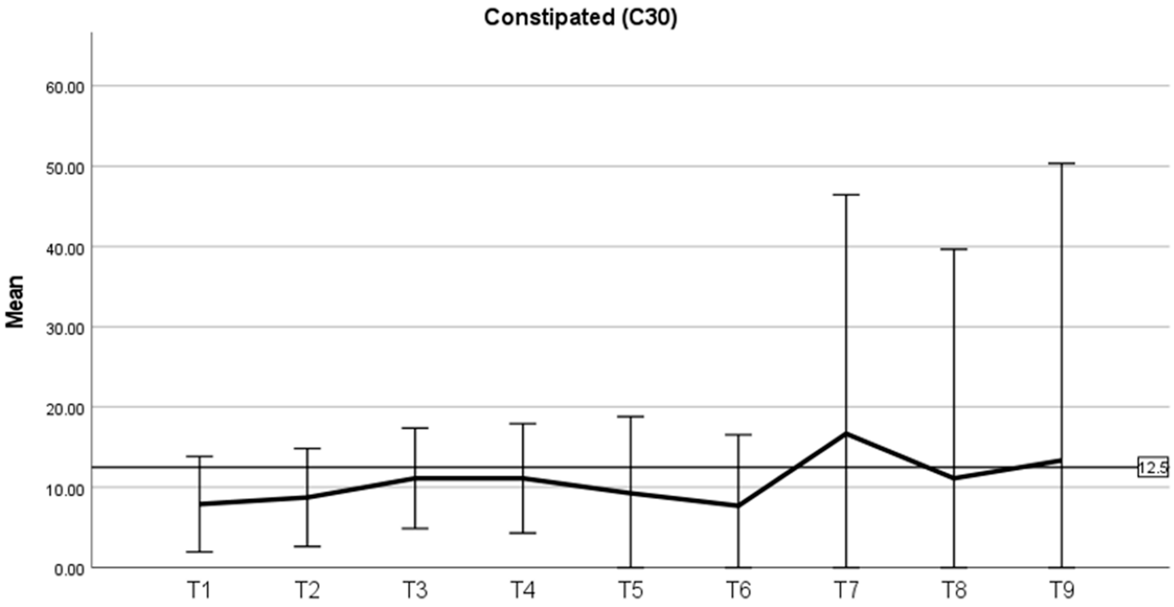
Constipated (C30)
Mean
60.00
50.00
40.00
30.00
20.00
10.00
0.00
12.5
T1
T2
T3
T4
T5
T6
T7
T8
T9

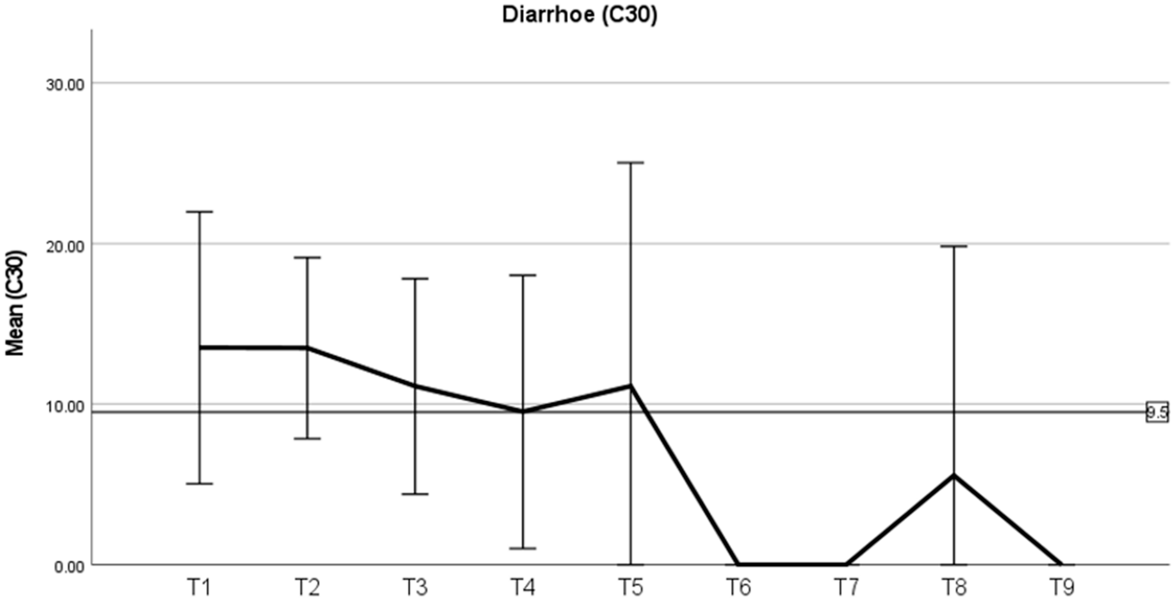
Diarrhoe (C30)
Mean (C30)
30.00
20.00
10.00
0.00
9.5
T1
T2
T3
T4
T5
T6
T7
T8
T9

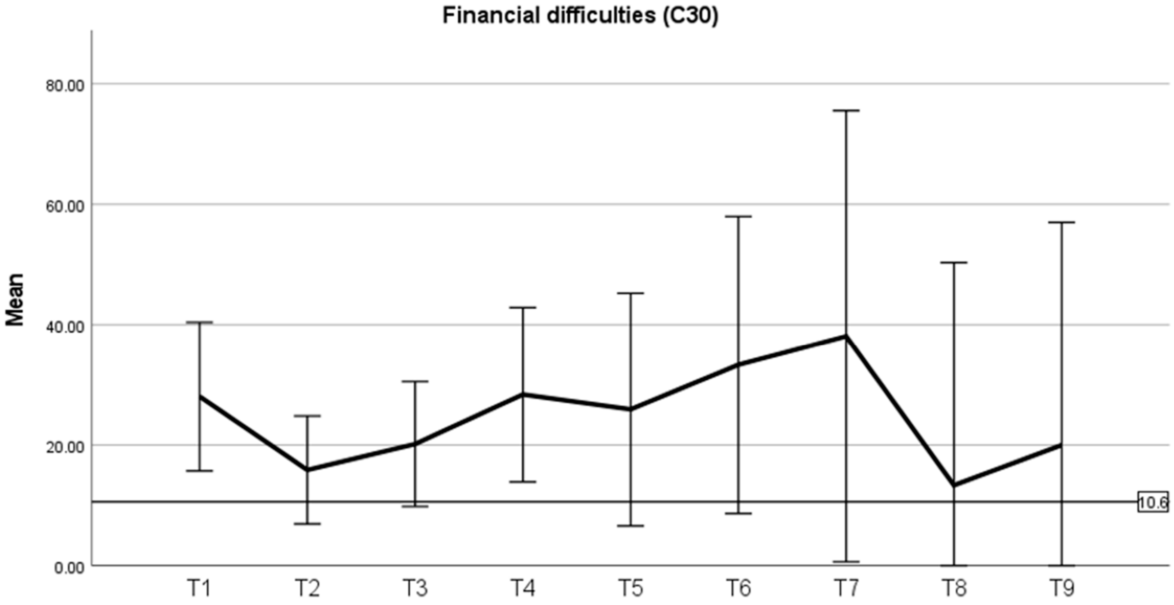
Financial difficulties (C30)
Mean
80.00
60.00
40.00
20.00
0.00
10.6
T1
T2
T3
T4
T5
T6
T7
T8
T9

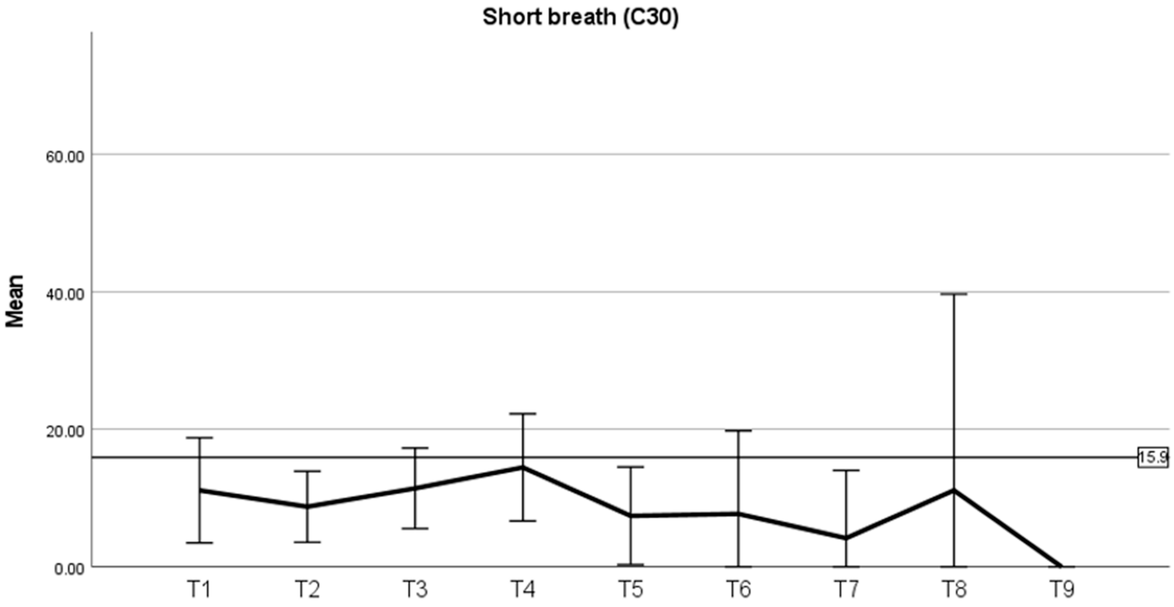
Short breath (C30)
Mean
60.00
40.00
20.00
0.00
15.9
T1
T2
T3
T4
T5
T6
T7
T8
T9

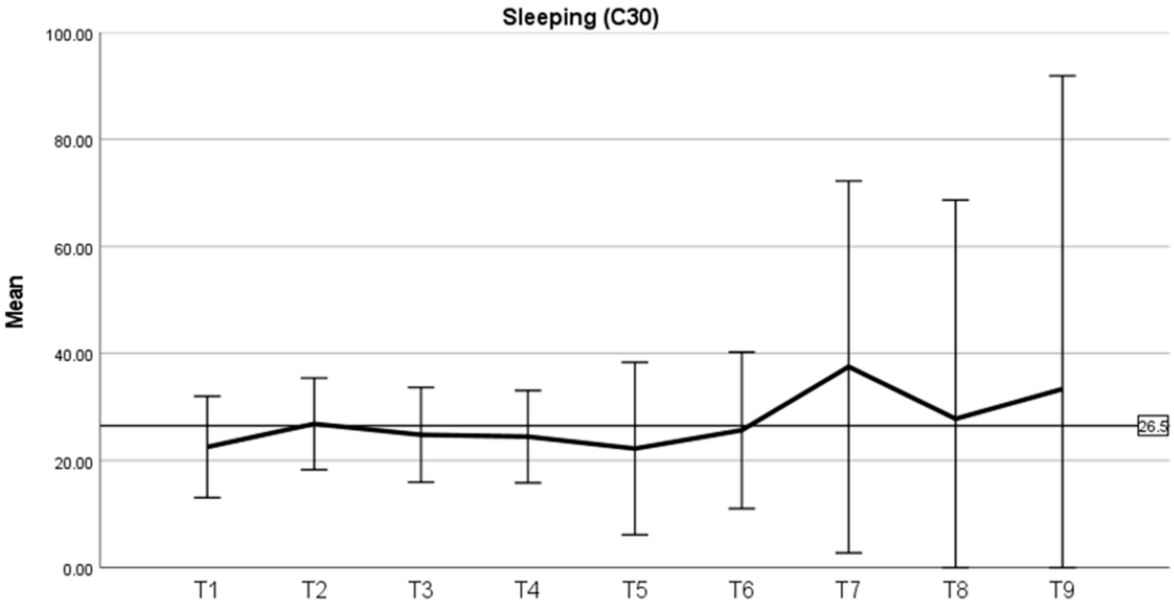
Sleeping (C30)
Mean
100.00
80.00
60.00
40.00
20.00
0.00
26.5
T1
T2
T3
T4
T5
T6
T7
T8
T9

EORTC QLQ-BN20

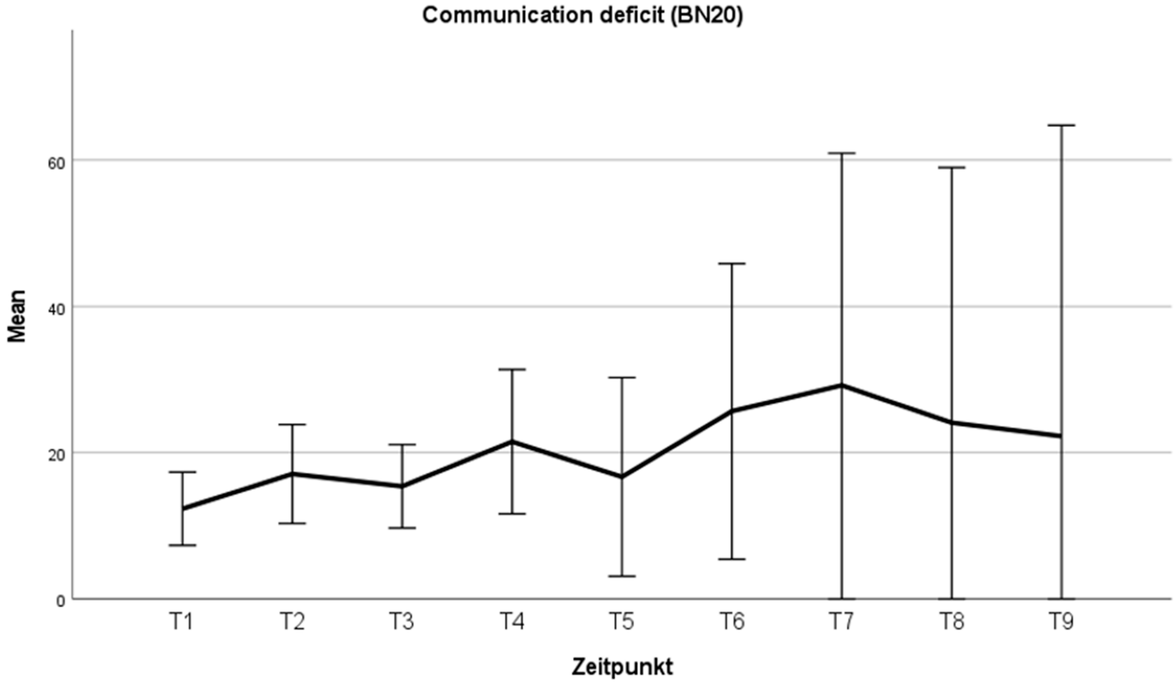


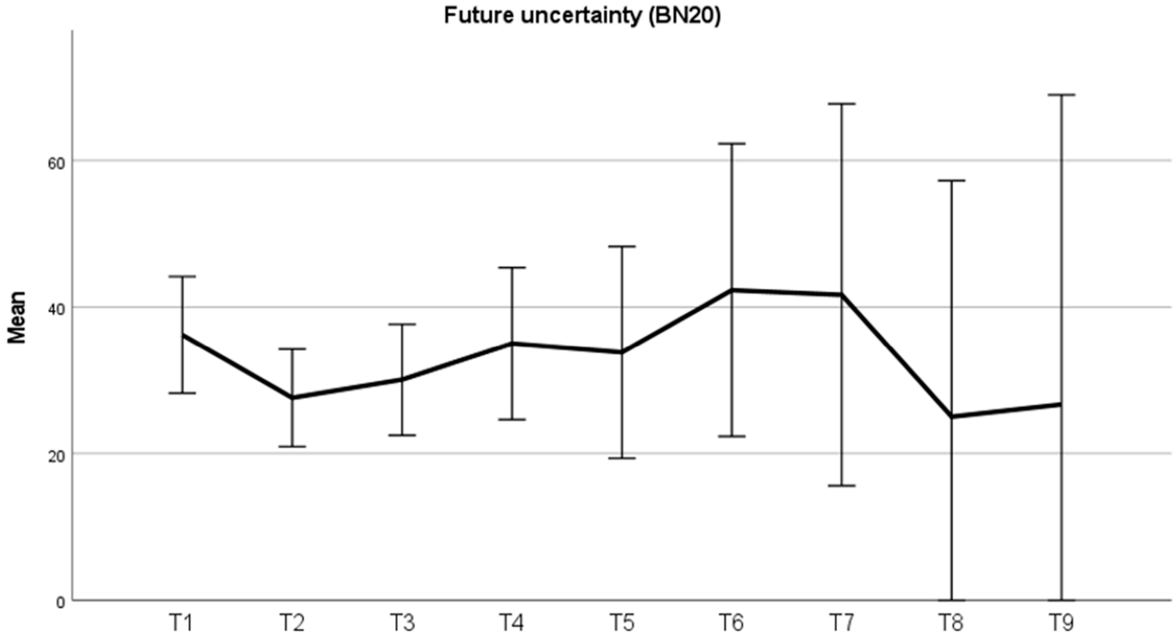

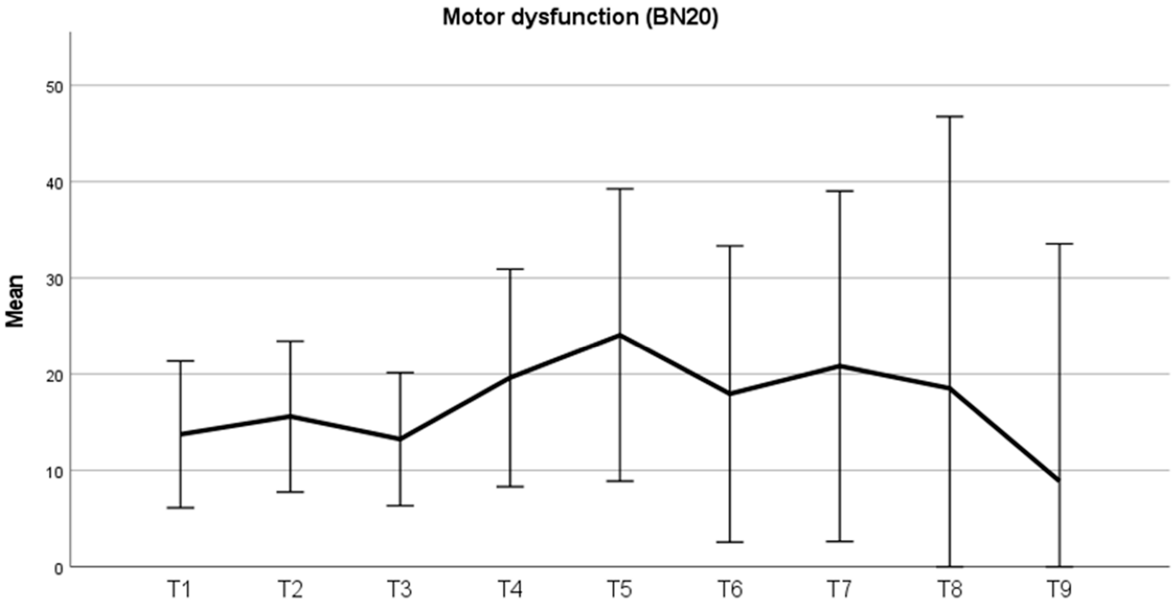
Motor dysfunction (BN20)
Mean
0
10
20
30
40
50
T1
T2
T3
T4
T5
T6
T7
T8
T9

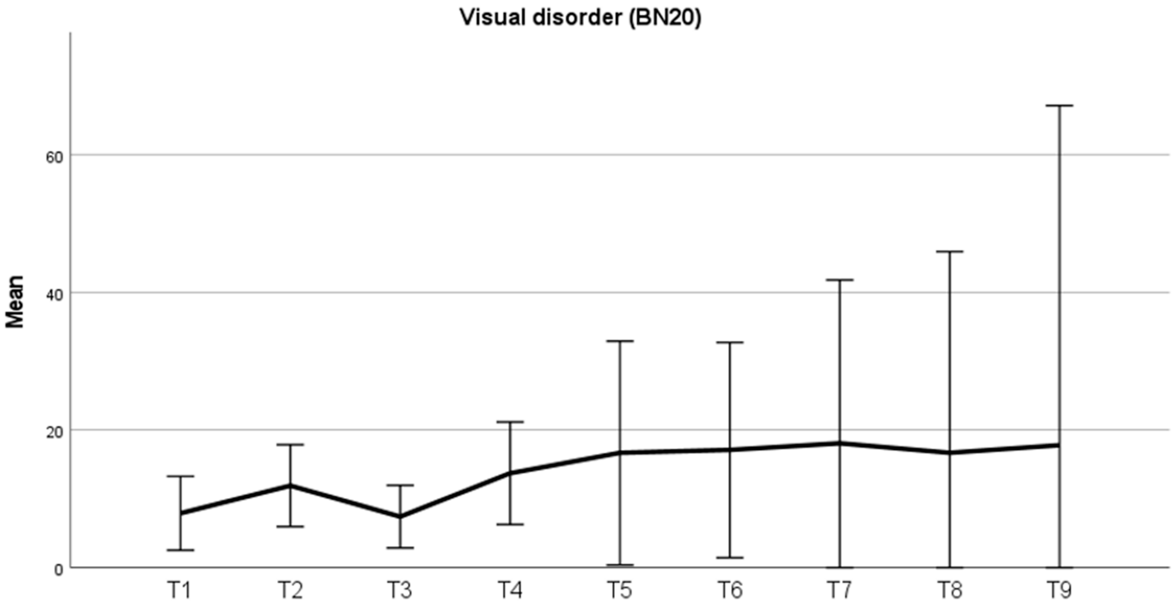
Visual disorder (BN20)
Mean
0
20
40
60
T1
T2
T3
T4
T5
T6
T7
T8
T9

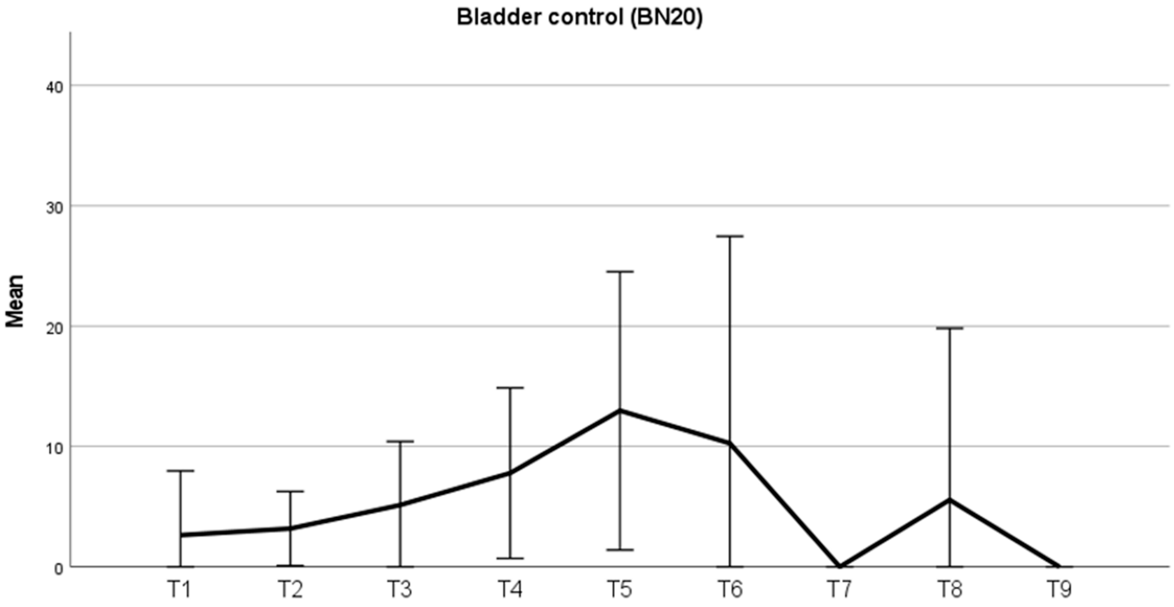
Bladder control (BN20)
Mean
0
10
20
30
40
T1
T2
T3
T4
T5
T6
T7
T8
T9

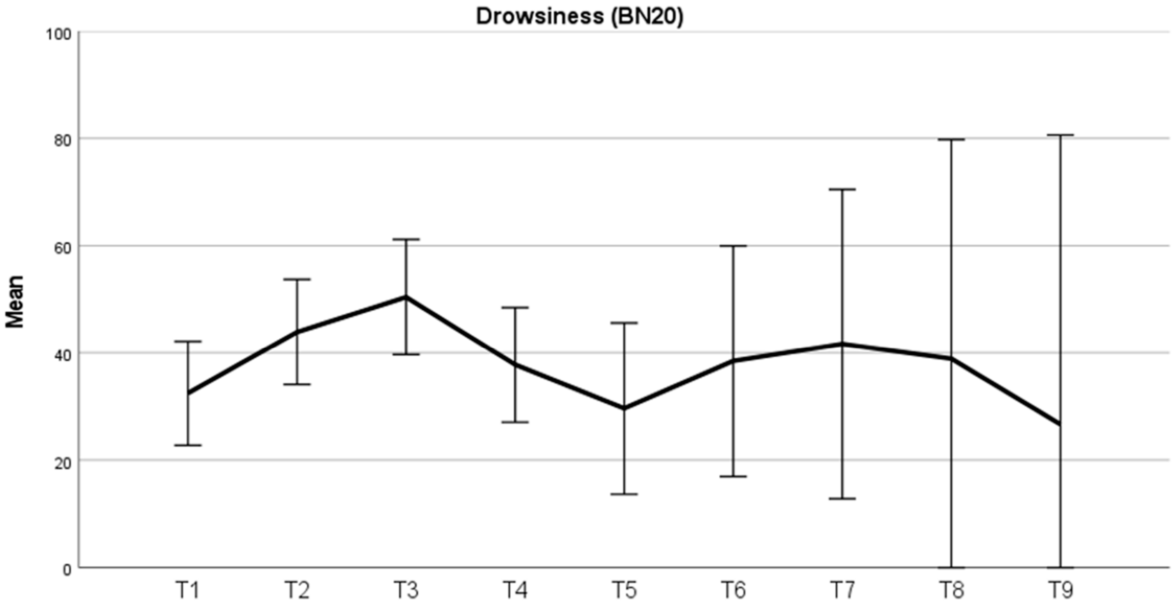
Drowsiness (BN20)
Mean
0
20
40
60
80
100
T1
T2
T3
T4
T5
T6
T7
T8
T9

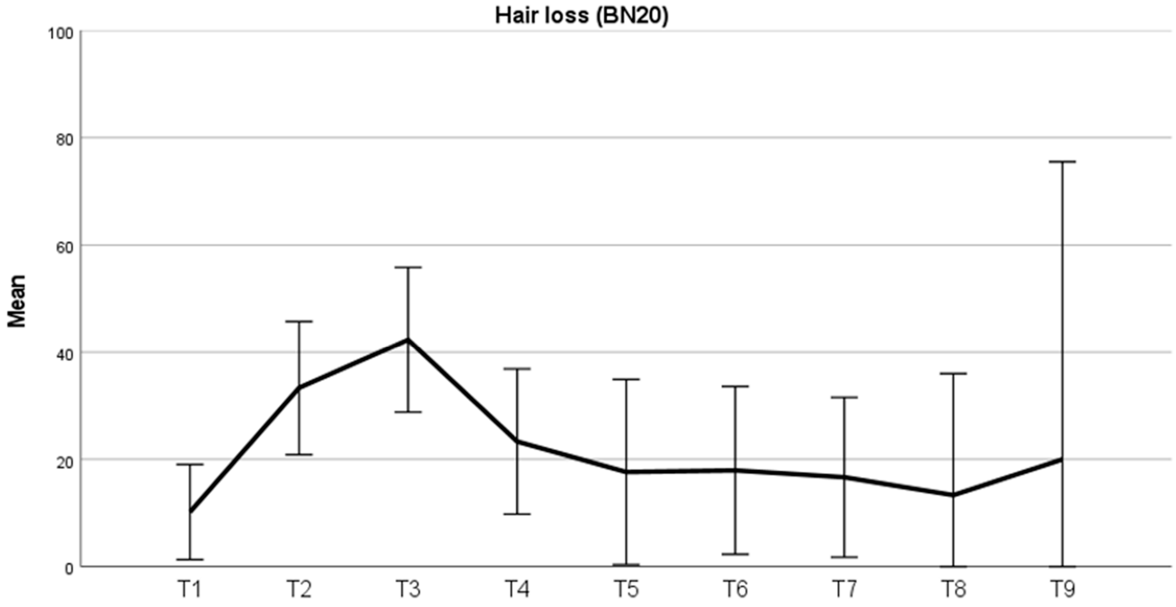
Hair loss (BN20)
Mean
0
20
40
60
80
100
T1
T2
T3
T4
T5
T6
T7
T8
T9

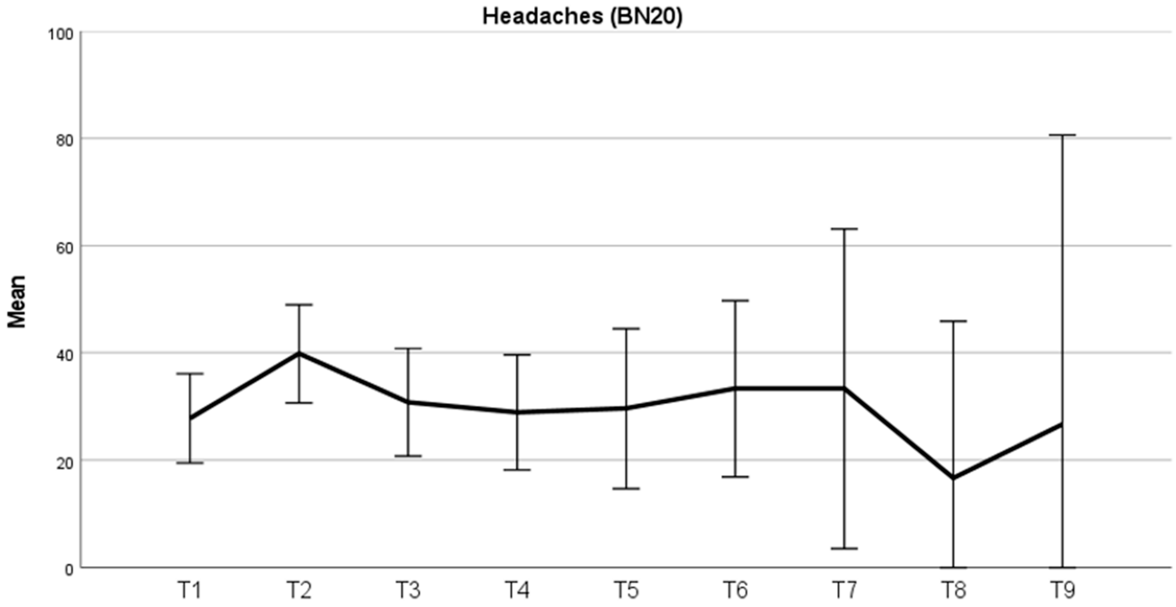
Headaches (BN20)
Mean
0
20
40
60
80
100
T1
T2
T3
T4
T5
T6
T7
T8
T9

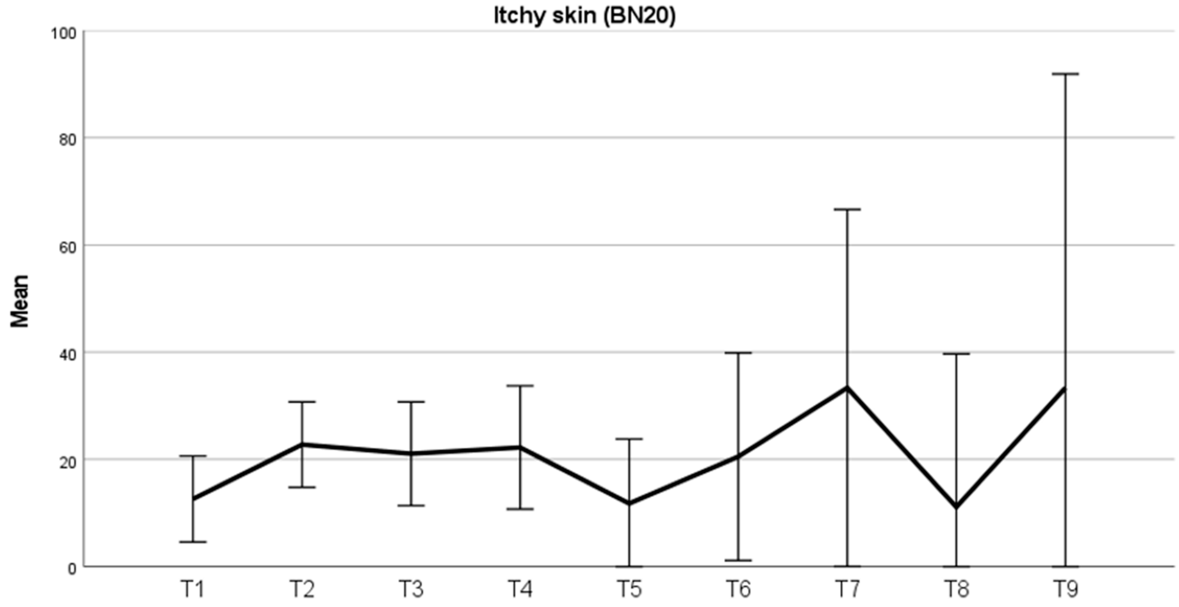
Itchy skin (BN20)
Mean
0
20
40
60
80
100
T1
T2
T3
T4
T5
T6
T7
T8
T9

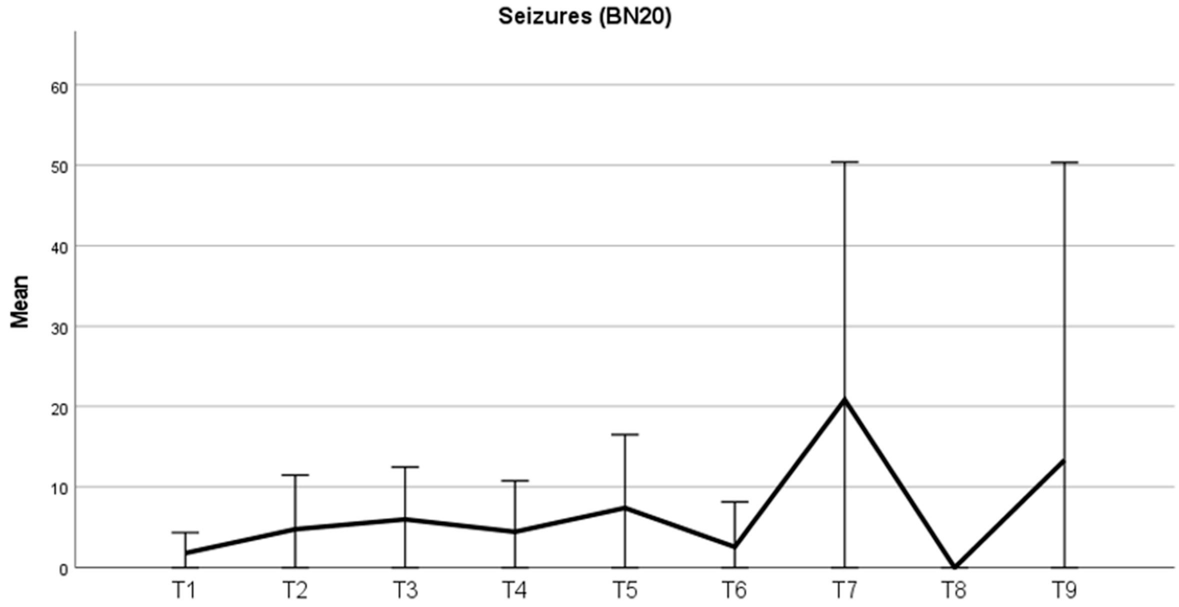
Seizures (BN20)
Mean
0
10
20
30
40
50
60
T1
T2
T3
T4
T5
T6
T7
T8
T9

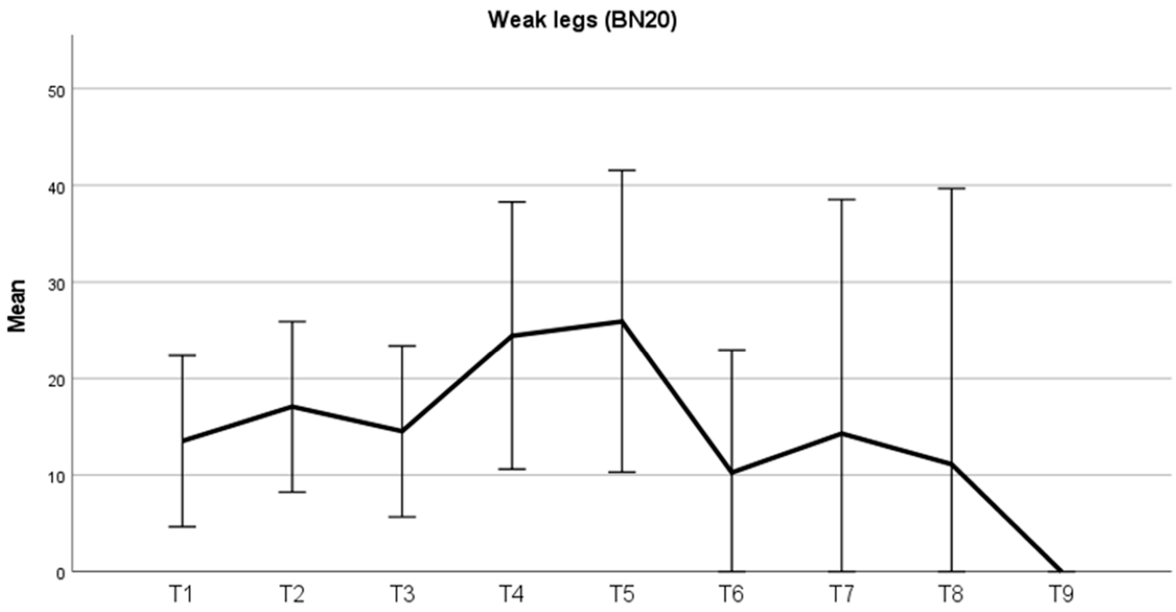
Weak legs (BN20)
Mean
50
40
30
20
10
0
T1
T2
T3
T4
T5
T6
T7
T8
T9
